# Metabolomic profiling reveals salivary hypotaurine as a potential early detection marker for medication-related osteonecrosis of the jaw

**DOI:** 10.1371/journal.pone.0220712

**Published:** 2019-08-12

**Authors:** Wakako Yatsuoka, Takao Ueno, Kanako Miyano, Yasuhito Uezono, Ayame Enomoto, Miku Kaneko, Sana Ota, Tomoyoshi Soga, Masahiro Sugimoto, Toshikazu Ushijima

**Affiliations:** 1 Dental Division, National Cancer Center Hospital, Tsukiji, Chuo-ku, Japan; 2 Course of Advanced Clinical Research of Cancer, Juntendo University Graduate School of Medicine, Hongo, Bunkyo-ku, Tokyo, Japan; 3 Division of Cancer Pathophysiology, National Cancer Center Research Institute, Tsukiji, Chuo-ku, Japan; 4 Division of Supportive Care Research, Exploratory Oncology Research and Clinical Trial Center, National Cancer Center, Tsukiji, Chuo-ku, Japan; 5 Innovation Center for Supportive, Palliative and Psychosocial Care, National Cancer Center Hospital, Tsukiji, Chuo-ku, Japan; 6 Institute for Advanced Biosciences, Keio University, Tsuruoka City, Yamagata, Japan; 7 Research and Development Center for Minimally Invasive Therapies Health Promotion and Preemptive Medicine, Tokyo Medical University, Shinjuku, Japan; 8 Division of Epigenomics, National Cancer Center Research Institute, Tsukiji, Chuo-ku, Japan; University of Florida, UNITED STATES

## Abstract

Medication-related osteonecrosis of the jaw (MRONJ) is a rare but serious adverse event of bone-modifying agents used to prevent bone complications in cancer patients with bone metastasis. Currently, early treatment is the only way to prevent further progression, as the pathogenesis of MRONJ has not yet been elucidated, and a standard treatment has not been established. The aim of this study was to identify a marker for early detection marker of MRONJ by exploring substances in saliva specific to MRONJ at an early stage. We collected salivary samples from 17 patients with MRONJ and conducted metabolomic analyses using capillary electrophoresis mass spectrometry for non-targeted analysis of hydrophilic metabolites. In the screening cohort, we compared the saliva of patients with stage ≥1 advanced MRONJ (*n* = 9) with that of controls without MRONJ before chemotherapy (*n* = 9). The top 5 most elevated salivary metabolites were histamine, 3-(4-hydroxyphenyl)propionate, malonate, carnosine, and hypotaurine. In the validation cohort, we analyzed additional patients with stage ≥1 advanced MRONJ (*n* = 8) and controls without MRONJ after chemotherapy (*n* = 9), confirming a significant 2.28-fold elevation in the salivary concentration of hypotaurine. These results revealed elevated salivary hypotaurine concentration as a potential marker for the early detection of MRONJ.

## Introduction

Medication-related osteonecrosis of the jaw (MRONJ) is a rare but serious adverse event in cancer patients treated with bone-modifying agents (BMAs), such as bisphosphonate preparations and anti-receptor activator of NF-kappaB ligand antibodies, for the prevention of skeletal-related events in cancer patients with bone metastases [1, 2]. Although the use of BMAs has greatly contributed to improving the quality of life (QOL) of patients with cancer bone metastasis, the occurrence of MRONJ can significantly impair QOL. The onset mechanism of MRONJ has not yet been clarified. Although surgical treatment is effective for stage 2 MRONJ [3, 4], appropriate treatment has not been established for stage 0 and 1 MRONJ [5, 6]. Therefore, intervention at an early stage is critical for avoiding devastating outcomes.

However, the initial stage of MRONJ (stage 0) is difficult to diagnose based on clinical findings. Additionally, >50% of patients with stage 0 MRONJ progress to stages 1 through 3 [7, 8]. The diagnostic criteria for MRONJ currently include only the following clinical findings: 1) current or previous treatment with an antiresorptive or antiangiogenic agent, 2) exposed bone or equivalent for longer than 8 weeks, and 3) no history of radiation therapy or obvious metastatic disease [1]. To date, only a limited number of reports have investigated candidate markers for the early detection of MRONJ. A study using ^18^F-NaF PET/CT reported that ^18^F-NaF uptake appeared useful for evaluating MRONJ [9]. Moreover, bone scintigraphic and radiographic features also accumulate in lesions at an early stage [10–12]; however, their usefulness in diagnosing MRONJ has not been established due to the requirement for complicated procedures involving X-ray exposure and high medical costs. Furthermore, no MRONJ-specific biomarker in biofluid has been reported.

Here, we isolated candidate substances specific to MRONJ by metabolomic analysis of patient saliva in order to identify substances potentially capable of detecting MRONJ onset. Saliva can be readily collected and can reflect various pathological conditions, making it attractive as a source for diagnosis of systemic diseases, such as oral cancer, breast cancer, and pancreatic cancer [13–15]. Additionally, abnormalities in bone metabolism might be a mechanism associated with MRONJ onset [1], with this activity possibly reflected in saliva. Metabolomic analysis allows comprehensive investigation of changes in metabolite type and concentration, thereby making it a more effective determinant of phenotype relative to other types of–omics analyses [16]. On the other hand, one disadvantage of metabolomic analysis is that unlike mutation or polymorphism analysis, it is susceptible to various external environmental factors, such as lifestyle.

## Materials and methods

### Study design and sample collection

**Fig 1:**
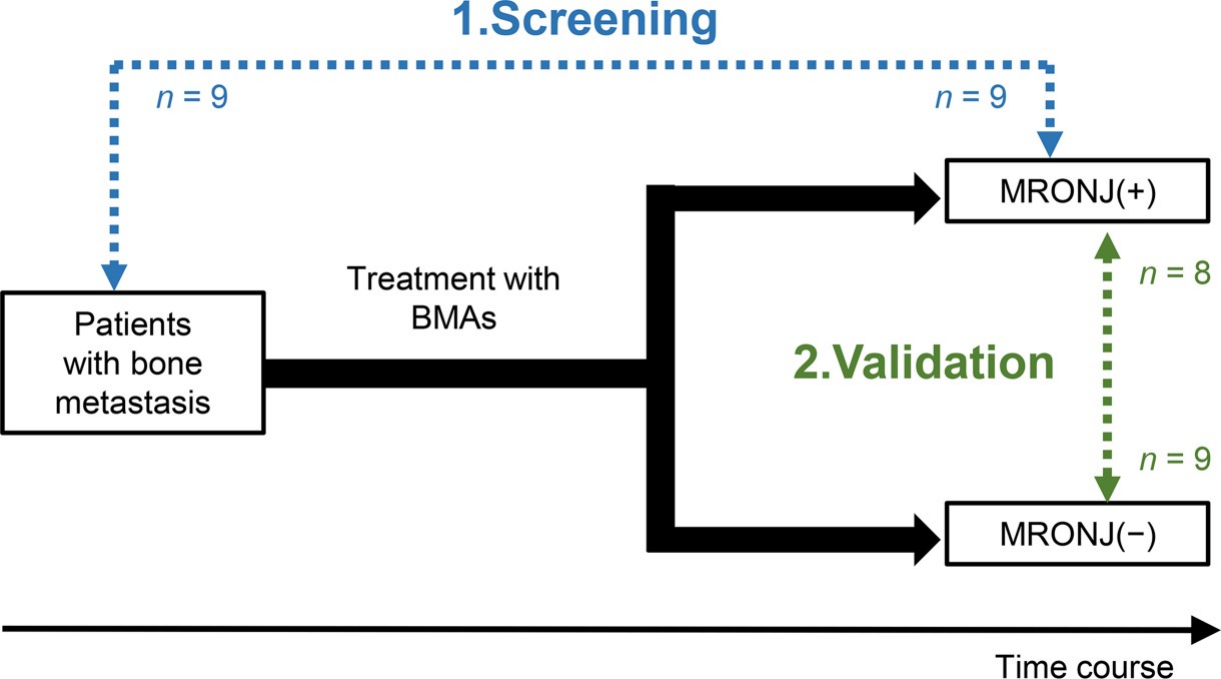
Study design. Open boxes represent datasets. In the screening cohort, nine patients with MRONJ stage ≥1 and nine patients before treatment with BMAs were compared by metabolomic analysis. In the validation cohort, eight patients with MRONJ stage ≥1 and nine patients without MRONJ after treatment with BMAs were compared in order to confirm the validity of findings in the screening cohort in a more clinically relevant setting.

A total of 35 patients with bone metastasis of solid cancer (regardless of cancer type) and being treated at the National Cancer Center Central Hospital were enrolled. This study was conducted in accordance with the Declaration of Helsinki. The screening cohort included nine patients with MRONJ stage ≥1 (5 males and 4 females; mean age: 70.8 ± 6.2 years) and nine patients not yet treated with BMAs and thus without MRONJ as a control group (5 males and 4 females; mean age: 61.7 ± 7.8 years). The validation cohort included eight patients with MRONJ stage ≥1 (4 males and 4 females; mean age: 69.0 ± 9.6 years) and nine patients without MRONJ after the use of BMAs as a control group (3 males and 6 females; mean age: 58.7 ± 8.7 years) (Fig 1). All participants have provided written informed consent before study enrollment. This study’s conduct was approved by National Cancer Center Ethics Committee (Approval Numbers 2016–068).

**Fig 2:**
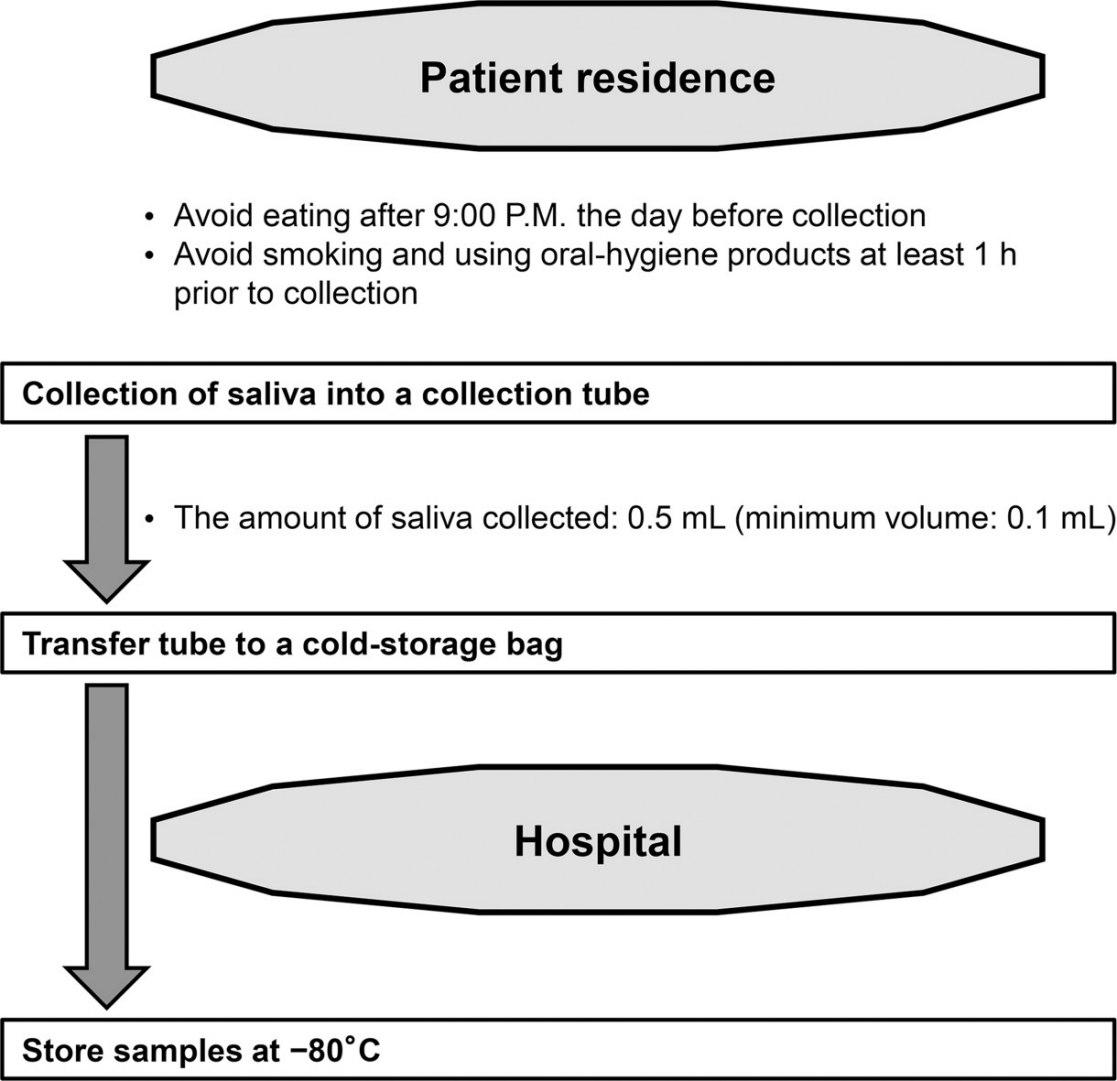
Sample collection. Patients provided a saliva sample following overnight fasting (~0.5 mL; minimum: 0.1 mL), transferred the sample to a cold-storage bag, and transported to the hospital within 4 h of collection, where it was stored at −80°C until use.

Saliva was collected, as follows. Patients were provided a collection tube (50-cc Falcon tube; Corning Inc., Corning, NY, USA), refrigerant, and refrigerating bag. Patients avoided eating after 9:00 P.M. the day prior to collection, and saliva was collected on the following morning in order to balance participant convenience and an optimal fasting time (reportedly 12-h fasting after dinner [17]). Patients refrained from eating, drinking, smoking, or use of oral-hygiene products for at least 1 h prior to saliva collection. Patients were instructed to rinse their mouth with tap water and, 5-min later, to spit into the collection tube placed in a Styrofoam cup filled with crushed ice in water. They were instructed not to cough up mucus. The amount of saliva collected was ~0.5 mL (minimum: 0.1 mL), and the collection tube was transferred to a cold-storage bag within a few minutes and transported to the hospital within 1 h to 4 h, after which the sample was frozen at −80°C (Fig 2).

### Sample processing for metabolomic analysis

Frozen saliva was thawed at 4°C for ~1.5 h, followed by their being dissolved using a vortex mixer and filtered through a 5-kDa cutoff filter (Millipore, Billerica, MA, USA) via centrifugation at 9,100*g* for at least 2.5 h at 4°C. Each sample (45 μL) was then aliquoted into a 1.5-mL Eppendorf tube (Sigma-Aldrich, St. Louis, MO, USA) along with 2 mM methionine sulfone, 2-[N-morpholino]-ethanesulfonic acid, d-camphol-10-sulfonic acid, sodium salt, 3-aminopyrrolidine, and trimesate.

### Capillary electrophoresis mass spectrometry (CE-MS) conditions

#### Instrument parameters

The metabolite standards, instrumentation, and CE time-of-flight (TOF) MS conditions were used according to previously described methods [18]. We performed CE-MS measurement as a single experiment for each sample. Due to the high cost of metabolomic analysis, most studies use only a single experiment. Additionally, a previous study indicated that interday and circadian variations influence study reproducibility more than analytical accuracy [19]. We performed cation analysis using an Agilent CE system (G1600AX), Agilent G1969A LC/MSD TOF system, Agilent 1100 series isocratic HPLC pump, G3251A Agilent CEMS adapter kit, and G1607A Agilent CE-electrospray ionization (ESI)-MS sprayer kit (Agilent Technologies, Santa Clara, CA, USA). We then performed anion analysis using the same equipment used for cation analysis. For cation and anion analyses, the CE-MS adapter kit included a capillary cassette that facilitates thermostatic control of the capillary. The CE-ESI-MS sprayer kit simplifies coupling of the CE system with the MS system and is equipped with an electrospray source. For system control and data acquisition, we used Agilent ChemStation software for CE (A10.02) and Agilent MassHunter software for TOF-MS (B.02.00). For anion analysis, we replaced the original Agilent SST316Ti stainless steel ESI needle with a passivated SST316Ti stainless steel and platinum needle (Agilent Technologies) passivated with 1% formic acid and a 20% aqueous solution of isopropanol at 80°C for 30 min for anion analysis.

Sample separation was performed in fused silica capillaries (50-μm i.d. × 100 cm total length) filled with 1 M formic acid as the running electrolyte for cationic metabolite analysis using CE-TOF-MS. Before the first use and for 4 min for tissue-sample analyses, the capillary was flushed before each sample injection with formic acid (1 M) for 20 min. For saliva sample analysis, we flushed the capillary with ammonium formate for 5 min, Milli-Q water for 5 min, and run buffer for 5 min. Sample solutions (~3 nL) were injected at 50 mbar for 5 s, and a voltage of 30 kV was applied. The temperature of the sample tray was kept at <5°C, and the capillary temperature was maintained at 20°C. The sheath liquid comprising methanol/water (50%, v/v) and 0.1 μM hexakis(2,2-difluoroethoxy) phosphazene (Hexakis) was delivered at 10 μL/min. We conducted ESI-TOF-MS in positive-ion mode and we set the capillary voltage at 4 kV, and the flow rate of nitrogen gas (heater temperature = 300°C) at 7 psig. For TOF MS, the fragmentor, skimmer, and OCT RF voltages were 75 V, 50 V, and 125 V, respectively. We performed automatic recalibration of each acquired spectrum using reference standards: [^13^C isotopic ion of protonated methanol dimer (2MeOH + H)]^+^, *m/z* 66.0631; and [protonated Hexakis (M + H)]^+^, *m/z* 622.0290. Mass spectra were acquired at a rate of 1.5 cycles/s over an *m/z* range of 50 to 1,000.

For anionic metabolite analysis using CE-TOF-MS, we used a commercially available Cosmo(+) capillary (50 μm × 105 cm; Nacalai Tesque, Kyoto, Japan) chemically coated with a cationic polymer for separation. Ammonium acetate solution (50 mM; pH 8.5) was used as the electrolyte for separation. The new capillary was flushed successively with the running electrolyte (pH 8.5), 50 mM acetic acid (pH 3.4) before the first use, and then the electrolyte again for 10 min each. The capillary was equilibrated for 2 min by flushing with 50 mM acetic acid (pH 3.4) before each injection and then with the running electrolyte for 5 min. A sample solution (~30 nL) was injected at 50 mbar for 30 s, and we applied a voltage of −30 kV. The sample tray was cooled to <5°C, and the capillary temperature was maintained at 20°C. To deliver 10 L/min of 5 mM ammonium acetate in 50% (v/v) methanol/water containing 0.1 M Hexakis to the CE interface, an Agilent 1100 series pump equipped with a 1:100 splitter (Agilent Technologies) was used. Here, it was used as a sheath liquid surrounding the CE capillary to provide a stable electrical connection between the tip of the capillary and the grounded electrospray needle. ESI-TOF-MS was conducted in negative-ionization mode at a capillary voltage of 3.5 kV. For TOF MS, the fragmentor, skimmer and OCT RF voltages were set at 100 V, 50 V, and 200 V, respectively. We maintained the flow rate of the drying nitrogen gas (heater temperature = 300°C) at 7 psig. Automatic recalibration of each acquired spectrum was done with reference standards: [^13^C isotopic ion of deprotonated acetic acid dimer (2 CH_3_COOH–H)]^–^, *m/z* 120.03841; and [Hexakis deprotonated acetic acid (M + CH_3_COOH–H)]^–^, *m/z* 680.03554. Exact mass data were acquired at a rate of 1.5 spectra/s over an *m/z* range of 50 to 1,000.

#### Processing of metabolomic data

We analyzed CE-TOF-MS raw data with our proprietary software, MasterHands. The peaks were identified by matching corresponding *m/z* values and normalized migration times to those of standard compounds. We calculated metabolite concentration based on the combination of a mixture of standard compounds with mixed internal standards.

### Statistical analysis

Statistical analyses were performed using the GraphPad Prism software (v.6.0; GraphPad Software, San Diego, CA, USA) and BellCurve for Excel (Social Survey Research Information Co., Ltd., Tokyo, Japan). Patient gender between groups was compared using a chi-squared test, and age was compared by Kruskal–Wallis test, followed by Dunn’s multiple comparisons test. Concentrations of metabolites between non-MRONJ and MRONJ patients were compared using the Student’s *t* test. For comparison of hypotaurine concentrations according to MRONJ stage, the Kruskal–Wallis test, followed by Dunn’s multiple comparisons test was used. Metabolite concentrations were normalized to those of amino acids. Differences with a p < 0.05 were considered statically significant.

## Results

### Overview of profiled metabolites

**Fig 3:**
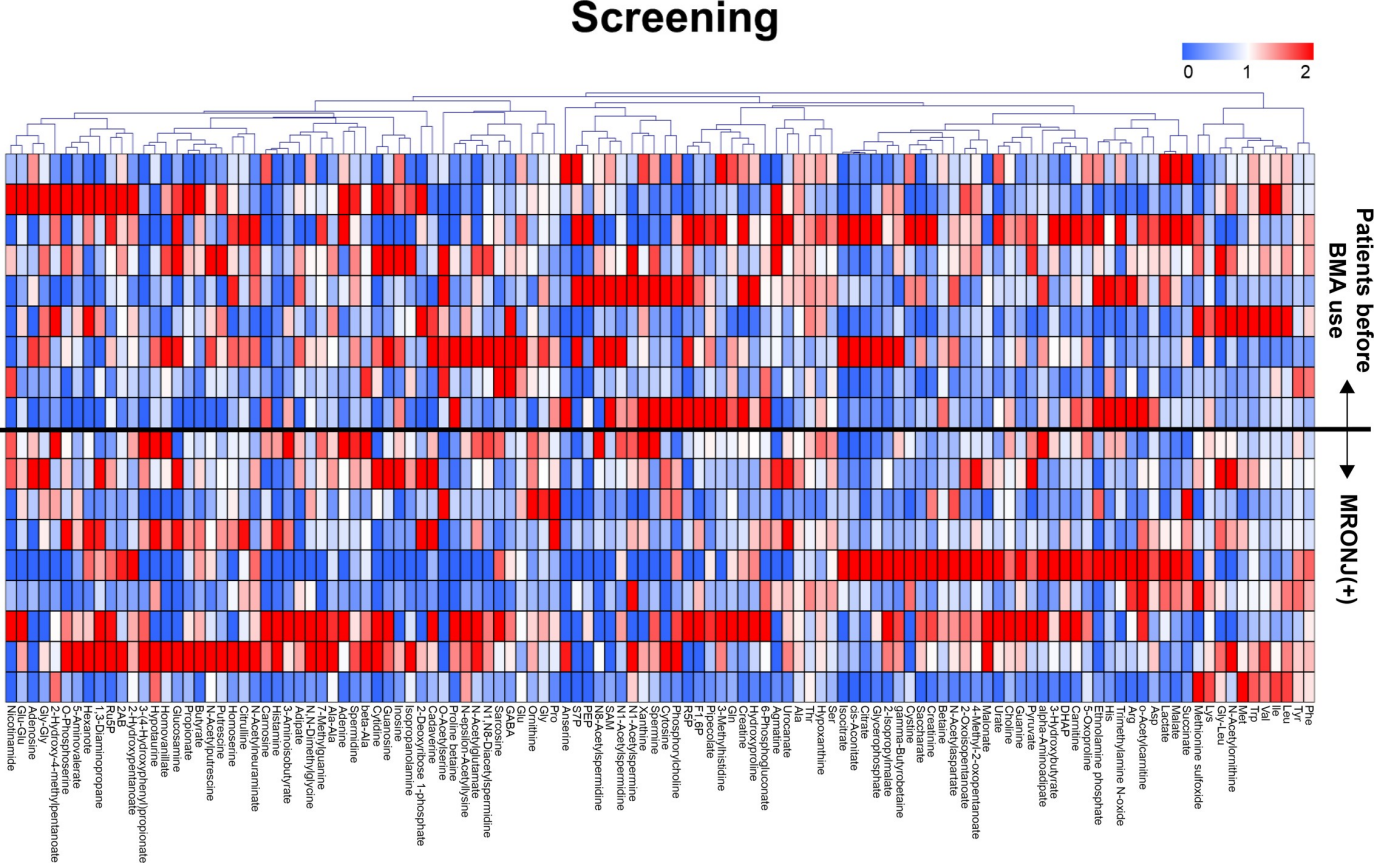
Heat map of salivary metabolomic profiles. Nine saliva samples from patients before BMA and nine from patients with MRONJ were analyzed by CE-MS. Of the 232 metabolites analyzed, 118 were detected in ≥50% of samples and used for supervised cluster analysis to separate the two groups. Color codes: red, high; blue, low; and white, average.

Exhaustive analysis of metabolites by CE-MS enabled identification and quantification of 232 metabolites. Among these, 118 metabolites were detected at a high frequency of ≥50% in the specimens. Because the majority of metabolites were detected in a small number of samples, we used cut-off values of 40%, 50%, and 60%; however, the results were essentially the same. Fig 3 shows a heat map allowing visualization of metabolite concentrations relative to one another using color coding.

### Screening of MRONJ-specific metabolite characteristics

**Fig 4:**
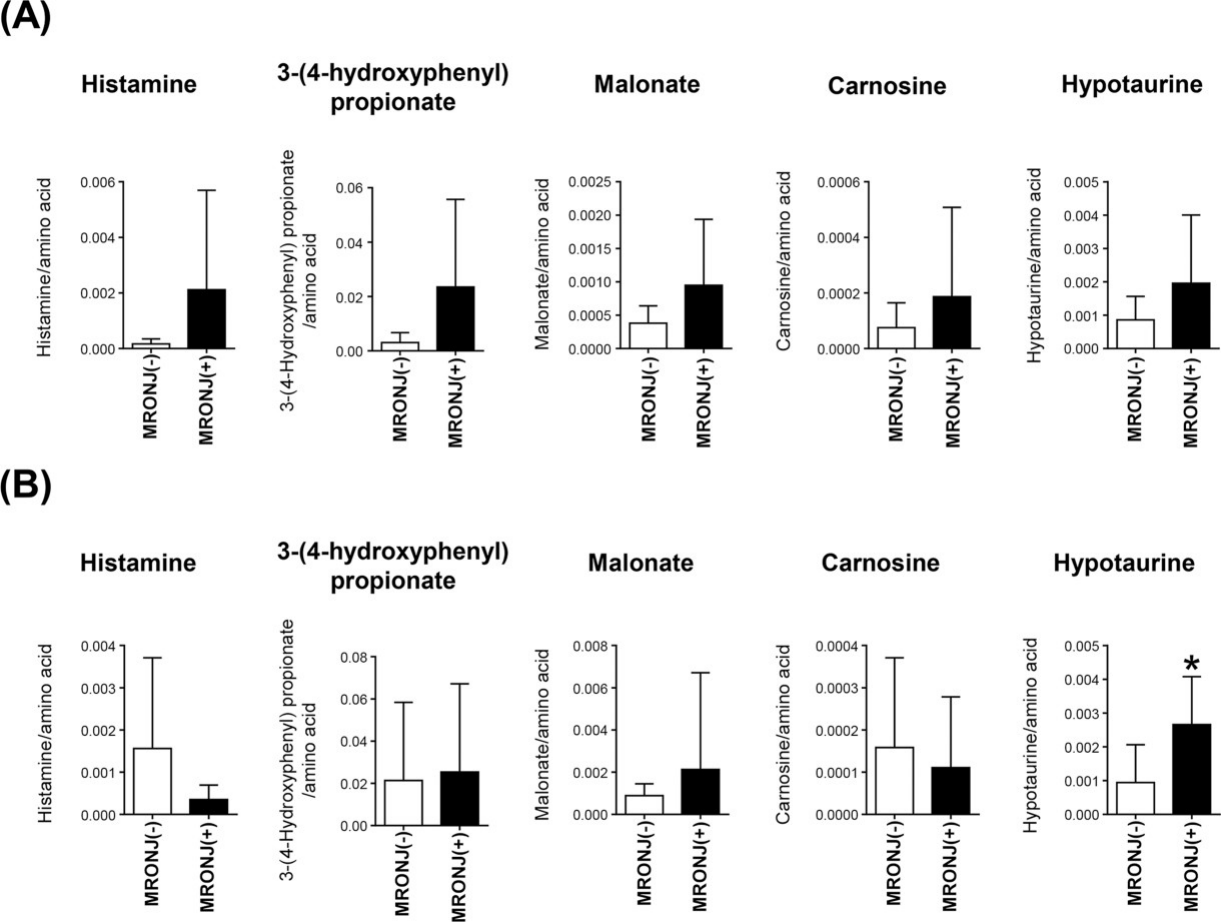
Candidate metabolites in the two cohorts. Five metabolites, including histamine, 3-(4-hydroxyphenyl)propionate, malonate, carnosine, and hypotaurine, showed the highest concentrations in the MRONJ group in the (A) screening cohort. In the (B) validation cohort, hypotaurine [MRONJ(−) vs MRONJ(+), 9.4 ± 11.2 × 10^−4^ vs. 26.6 ± 14.2 × 10^−4^; p = 0.017] and malonate (8.9 ± 5.6 × 10^−4^ vs. 21.3 ± 45.8 × 10^−4^; p = 0.472) showed consistently higher concentrations in MRONJ patients, although only hypotaurine was significantly higher. *p < 0.05.

The screening aimed to identify metabolites potentially associated with MRONJ. For this analysis, we compared the saliva samples of nine patients with demonstrated stage ≥1 MRONJ (5 males and 4 females) with those from nine patients (5 males and 4 females) before the use of BMAs (and thus without MRONJ). Patient sex and age did not differ between the two groups (Table 1). Among the 118 metabolites considered, the greatest differences between the two patient groups were observed for histamine [MRONJ(−) vs. MRONJ(+): 1.7 ± 1.8 × 10^−4^ (mean ± SD) vs. 21.2 ± 35.7 × 10^−4^; p = 0.141], 3-(4-hydroxyphenyl)propionate (30.7 ± 36.6 × 10^−4^ vs. 235.3 ± 321.9 × 10^−4^; p = 0.094), malonate (3.8 ± 2.6 × 10^−4^ vs. 9.5 ± 9.9 × 10^−4^; p = 0.129), carnosine (0.8 ± 1.9 × 10^−4^ vs. 1.9 ± 3.2 × 10^−4^; p = 0.342), and hypotaurine (8.6 ± 7.0 × 10^−4^ vs. 19.6 ± 20.4 × 10^−4^; p = 0.159) (Fig 4).

**Table 1:** Patient characteristics.

	Non-MRONJ patients		MRONJ patients	
Characteristic	Screening	Validation	Screening	Validation
**Description**	Before treatment with BMAs	Without MRONJ after treatment with BMAs	Stage ≥1	Stage ≥1
**Age**				
**Min–Max (median)**	49–70 (61)	45–70 (64)	62–80 (70)	61–80 (74)
**Sex**				
**Male**	5	3	5	4
**Female**	4	6	4	4
**Total**	9	9	9	8

There was no significant difference in age or sex between the non-MRONJ and MRONJ groups.

### Validation of candidate metabolites

To rule out false-positive results due to multiple testing of the 118 metabolites, we performed validation analysis to identify metabolites capable of distinguishing patients with and without MRONJ among those treated with BMAs. We used the saliva samples of eight patients (4 males and 4 females) with stage ≥1 MRONJ and nine non-MRONJ patients (3 males and 6 females) after the use of BMAs. Again, neither sex nor age differed between the two groups (Table 1). Two of the five candidate metabolites identified in the screening cohort, hypotaurine [MRONJ(−) vs MRONJ(+), 9.4 ± 11.2 × 10^−4^ vs. 26.6 ± 14.2 × 10^−4^; p = 0.017] and malonate (8.9 ± 5.6 × 10^−4^ vs. 21.3 ± 45.8 × 10^−4^; p = 0.472), showed consistently higher concentrations in MRONJ patients, although only hypotaurine was significantly higher.

**Fig 5:**
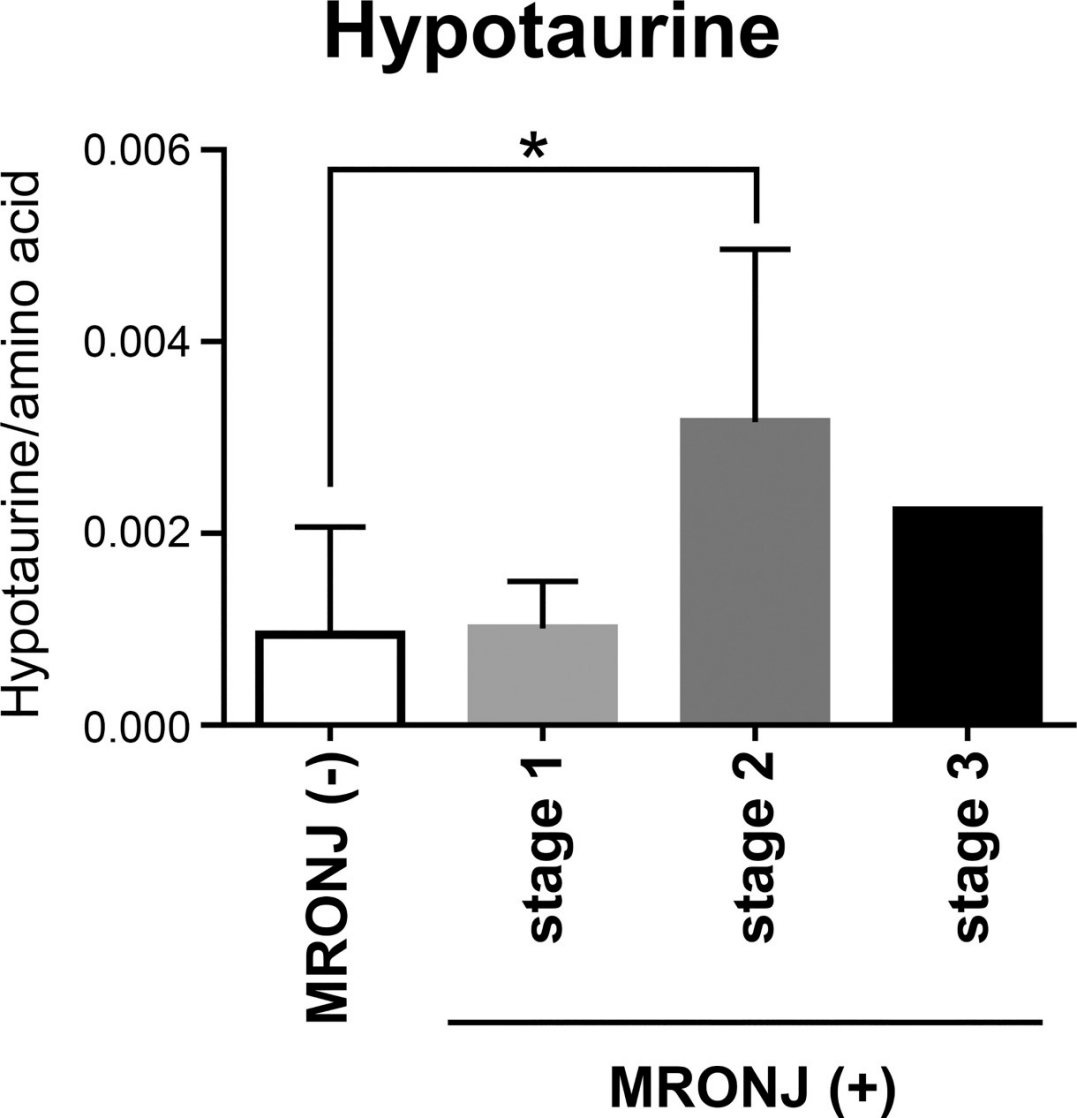
Relationships between hypotaurine concentration and MRONJ stage. Hypotaurine concentrations were analyzed in specimens from the screening and validation patient cohorts. The graph shows data representing the mean ± SD. The open and filled bar graphs show MRONJ(−) and MORONJ(+), respectively. There was a significant difference in hypotaurine concentration between the control (*n* = 18) and stage 2 groups (*n* = 9) but not with the stage 1 group (*n* = 6) or stage 3 group (*n* = 2). *p < 0.05.

We then stratified patients with MRONJ by stage in order to evaluate hypotaurine concentrations (Fig 5). Patients with stage 2 disease had a significantly higher concentration of hypotaurine relative to those in the control group, whereas those with stage 1 disease did not. Because there were only two cases with stage 3 disease, statistical significance could not be assessed in this group.

## Discussion

Hypotaurine concentrations were significantly higher in the MRONJ group in the validation cohort; therefore, we consider this a candidate metabolite for the early detection of MRONJ. Hypotaurine is an intermediate in the normal synthesis of taurine [20] and can act as an antioxidant involved in cellular defense against oxidative stress [20–22]. Histamine is reportedly associated with allergies and inflammation [23], propionate is a major microbial fermentation metabolite present in the human gut and that provides putative health effects [24], and malonate induces mitochondrial stress [25]. Additionally, the therapeutic potential of carnosine supplementation is reportedly associated with clinical consequences in a number of diseases [26]. In the metabolic pathway of taurine, cysteine sulfinic acid is first synthesized from cysteine by cysteine dioxygenase, followed by decarboxylation by cysteine sulfinic acid decarboxylase to generate hypotaurine, which is oxidized to synthesize taurine. Taurine is a neutral substance and can therefore be difficult to measure quantitatively; however, as levels of hypotaurine increase, taurine levels also reportedly increase based on the taurine metabolic pathway [20]. The biological role of hypotaurine has not yet been fully clarified.

Taurine concentration in the saliva fluctuates during fatigue due to exercise. As fatigue progresses, the concentration of taurine increases, suggesting that an increased taurine concentration might be a biological response associated with maintaining bodily functions under the load induced by high-intensity exercise [27]. Additionally, a previous study reported elevated taurine levels in gingival crevicular fluid of patients with severe periodontal disease, with defense against inflammatory reactions considered a possible relevant mechanism [28]. MRONJ pathogenesis reportedly involves bacterial infection and inflammation [29, 30]. Moreover, the risk of developing MRONJ increases with tooth extraction; however, it could be that these teeth already harbor periodontal disease or apical periodontitis and, thus, are already exposed to inflammation and infection [31–33]. This suggests that the presence of MRONJ might only be identified upon tooth extraction. However, even before tooth extraction, increased hypotaurine concentration might be present as a biological response to the previous establishment of MRONJ. Our results indicated the possibility that elevated hypotaurine concentration is specific to MRONJ and correlated with disease stage. This suggests that elevated hypotaurine concentration might be efficacious for diagnosing early stage MRONJ. In addition, we also reported that no increase in hypotaurine concentration in some inflammation states such as in patients with periodontal disease and oral cancer [13]. Our future work, accordingly, will focus on investigating whether hypotaurine concentration is elevated by inflammation. In addition, we considered it valid to conduct metabolome analysis of the jaw bone samples from MRONJ patients.

Saliva collection in cancer patients was initially reported to be difficult due to the fact that pain caused by MRONJ or oral mucositis likely decreases saliva output [34]. Unexpectedly, sufficient amounts of saliva were collected from all patients enrolled in this study. Additionally, saliva quality needs to be controlled for accurate metabolomic analysis. A previous report suggested that saliva samples treated at room temperature need to have a preservation time of ≤30 min, and for long-term storage, samples should be stored at −18°C. Moreover, if the saliva samples are stored at room temperature or 4°C, ethanol and an internal standard should be added to the salivary samples [35]. In the present study, samples were collected by the patients; therefore, the validity of the collection procedures requires further investigation. Because this study highlights the potential efficacy of metabolomic analysis of saliva, it might be useful to develop devices for saliva collection and storage.

This study has several limitations. First, the number of patients enrolled was small. In particular, the number of patients with stage 3 MRONJ was too small for statistical analysis. Nevertheless, compared with recent reports using metabolomic analysis, including those using 32 oral squamous cell carcinoma patients [36], 12 Sjögren's syndrome patients [37], 22 oral cancer patients [17], the number of samples from MRONJ patients used in the present study (*n* = 17) was comparable to previous studies. Second, to identify potential biomarkers and provide data for future studies involving larger cohorts, the control groups used in the present study were not identical. In the screening cohort, patients were enrolled before the use of BMAs, whereas the validation cohort included patients without MRONJ after the use of BMAs. For further development of markers for the early diagnosis of MRONJ, saliva specimens from the same patient before and after the development of MRONJ will be most informative.

In conclusion, our results identified and validated hypotaurine as a candidate biomarker of MRONJ development. Further studies involving more patients are warranted.

## Conclusions

Our study identified and validated hypotaurine as a candidate biomarker of MRONJ development. Further studies involving more patients are warranted.
